# Inflammation and vitamin D: the infection connection

**DOI:** 10.1007/s00011-014-0755-z

**Published:** 2014-07-22

**Authors:** Meg Mangin, Rebecca Sinha, Kelly Fincher

**Affiliations:** Chronic Illness Recovery, Fort Worth, Texas, USA

**Keywords:** Vitamin D, Infection, Inflammation, Immunotherapy

## Abstract

**Introduction:**

Inflammation is believed to be a contributing factor to many chronic diseases. The influence of vitamin D deficiency on inflammation is being explored but studies have not demonstrated a causative effect.

**Methods:**

Low serum 25(OH)D is also found in healthy persons exposed to adequate sunlight. Despite increased vitamin D supplementation inflammatory diseases are increasing. The current method of determining vitamin D status may be at fault. The level of 25(OH)D does not always reflect the level of 1,25(OH)2D. Assessment of both metabolites often reveals elevated 1,25(OH)2D, indicating abnormal vitamin D endocrine function.

**Findings:**

This article reviews vitamin D's influence on the immune system, examines the myths regarding vitamin D photosynthesis, discusses ways to accurately assess vitamin D status, describes the risks of supplementation, explains the effect of persistent infection on vitamin D metabolism and presents a novel immunotherapy which provides evidence of an infection connection to inflammation.

**Conclusion:**

Some authorities now believe that low 25(OH)D is a consequence of chronic inflammation rather than the cause. Research points to a bacterial etiology pathogenesis for an inflammatory disease process which results in high 1,25(OH)2D and low 25(OH)D. Immunotherapy, directed at eradicating persistent intracellular pathogens, corrects dysregulated vitamin D metabolism and resolves inflammatory symptoms.

## Introduction

Inflammation is involved in many chronic diseases and concern has been raised about the influence of vitamin D deficiency on inflammatory processes. When studies found an association between inflammatory diseases and low serum 25-hydroxyvitamin D (25(OH)D), further research found evidence of low vitamin D in a large segment of the general population. This led some authorities to declare a world-wide epidemic of vitamin D deficiency and to recommend vitamin D supplementation. Experts are debating the definition of vitamin D deficiency and the appropriate vitamin D doses, while further research is being done to determine if vitamin D supplementation has the intended effect.

According to some current definitions of vitamin D deficiency, even healthy persons, exposed to adequate sunlight, are unable to acquire enough vitamin D without supplementation. Often reiterated causes of vitamin D deficiency can be disputed in the light of more current research. In the absence of definitive studies, authorities are questioning the wisdom of supplementing the general population with vitamin D. The definition of Vitamin D deficiency needs re-evaluation in view of the fact that low 25(OH)D is found in both healthy and sick individuals. Concerns about vitamin D deficiency merit a closer look at the current method of determining vitamin D status because the level of 25(OH)D does not always reflect the level of 1,25-dihydroxyvitamin-D (1,25(OH)2D). Analysis of this active metabolite may reveal elevated 1,25(OH)2D) in the presence of low 25(OH)D and lead to a diagnosis of abnormal vitamin D endocrine system function.

An infectious pathogenesis posits that intracellular bacteria disrupt the vitamin D regulated immune system, resulting in persistent infection and chronic inflammation. In the clinical setting, a novel immunotherapy is demonstrating the ability to resolve vitamin D metabolism dysfunction, restore immune function, and thus, eliminate infection and reduce inflammation. This review ponders the question, “Is low 25(OH)D a cause of, or a consequence of inflammation?” The answer is found in the evidence that adds persistent intracellular infection to the equation.

## Vitamin D metabolism

**Fig. 1:**
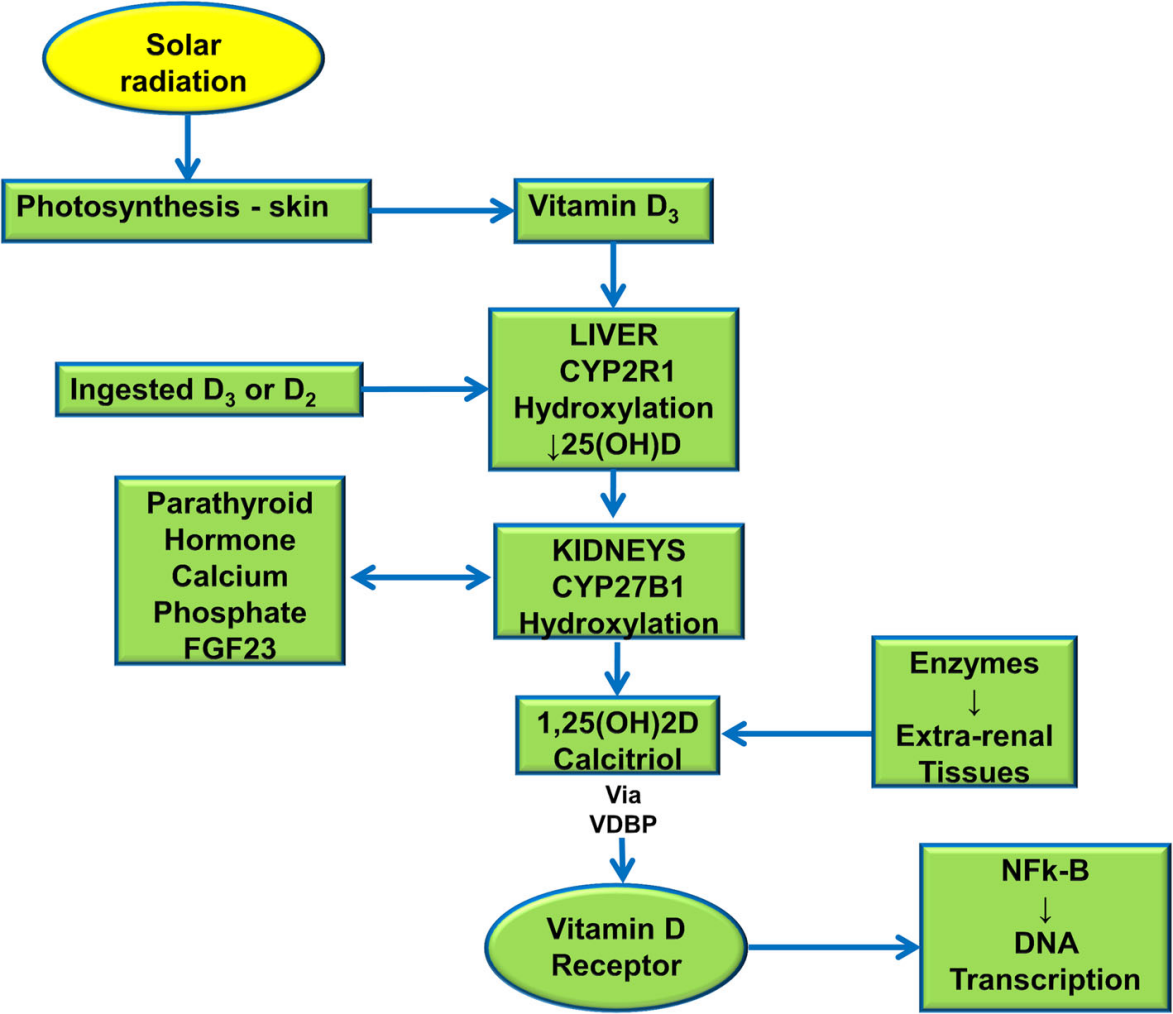
Synthesis and metabolism of vitamin D. Sequential metabolic processes convert biologically inactive, parental vitamin D into active metabolites

The sequential metabolic processes that convert biologically inactive, parental vitamin D into active metabolites begin when vitamin D_3_ is photosynthesized in the skin or when vitamin D_2_ or D_3_ is ingested. Vitamin D is transported to the liver where it is hydroxylated by an enzyme (CYP2R1, also known as cytochrome P450 2R1) to produce 25(OH)D [1]. 25(OH)D is then transported to the kidneys where it is hydroxylated by another enzyme (CYP27B1, formerly 1a-hydroxylase) to produce 1,25(OH)2D. 1,25(OH)2D (also known as calcitriol), the active metabolite, is the most potent steroid hormone in the human body [2]. Feedback mechanisms regulate production of 1,25(OH)2D in the kidneys via serum levels of parathyroid hormone (PTH), fibroblast-like growth factor-23 (FGF23) calcium, and phosphate [3]. 1,25(OH)2D is also produced in many other tissues (e.g., skin, macrophages, colon, pancreas, blood vessels, etc.) by enzymatic actions [4]. The vitamin D binding protein (VDBP) transports 1,25(OH)2D to the vitamin D receptor (VDR) in the cell nucleus [5]. The VDR is a member of the nuclear receptor family of ligand-regulated transcription factors. 1,25(OH)2D binds to the VDR and mediates the transcription of DNA, triggered by signaling proteins, like nuclear factor kappa-B (NFk-B) [6] (Fig. 1).

The influence of 1,25(OH)2D on the immune system is one of its most important roles. 1,25(OH)2D regulates the immune system via the VDR which is present in most immune cell types, particularly in antigen-presenting cells (APCs) such as monocytes, macrophages and dendritic cells [7]. 1,25(OH)2D activates the VDR to express antimicrobial peptides (AMPs) such as cathelicidin and beta defensins which attack pathogens [8, 9]. In general, the innate immune system is enhanced and the adaptive immune system is inhibited by 1,25(OH)2D [10, 11]. Thus, an effective immune response is heavily dependent on the vitamin D endocrine system which performs a balancing act of inflammation versus anti-inflammation.

## Vitamin D deficiency

Concerns about vitamin D deficiency arose when studies showed patients with autoimmune diseases have lower levels of serum 25(OH)D and study subjects given vitamin D had lower rates of autoimmune diseases and fewer markers of inflammation [12, 13]. However, authorities have not agreed on the significance of low 25(OH)D and without a consistent normal range for serum 25(OH)D, the definitions of vitamin D insufficiency and deficiency from the Vitamin D Council, the Endocrine Society and the Institute of Medicine vary significantly.

The Vitamin D Council definition [14]:

- Deficient: 0–40 ng/ml
- Sufficient: 40–80 ng/ml
- High normal: 80–100 ng/ml

The Endocrine Society definition [15]:

- Deficiency ≤ 20 ng/ml
- Insufficiency = 20–29 ng/ml

The Institute of Medicine definition [16]:

- Risk/deficiency ≤12 ng/ml
- Risk/insufficiency = 12–20 ng/ml
- Sufficient = 20 ng/ml

In 2006, the Merck Manual listed 25–40 ng/ml as the normal 25(OH)D range [17]. Recently, this range has skyrocketed to 30–74 ng/ml [18]. Quest Diagnostics now lists the upper limit of normal 25(OH)D as 100 ng/ml [19]. Laboratory reference ranges for serum 25(OH)D levels have long been based upon average values from populations of healthy individuals but many people are now supplementing with vitamin D. In the US, the leading authority regarding medical research is the prestigious Institute of Medicine (IOM). The 2010 IOM report on vitamin D emphasized that the current measurements, or cut-off points, of sufficiency and deficiency of 25(OH) D in use by laboratories have not been set using rigorous scientific studies. It suggests that since no central authority has determined which cut-off points to use, reports of deficiency and lab ranges may be skewed and numbers overestimated [16]. Therefore, it would be prudent to use the IOM vitamin D deficiency guideline in the clinical setting, for clinical studies and when evaluating research results.

## Purported reasons for vitamin D deficiency

Is low 25(OH)D among the general population an accurate assessment of vitamin D deficiency? Many reasons are cited for the current ‘epidemic’ of vitamin D ‘deficiency’ but closer examination reveals these beliefs are based on outdated or limited studies and can be challenged with more recent research.

Melanin pigmentation is only one factor that determines the amount of vitamin D_3_ which is photosynthesized [20, 21]. Bogh et al. [22] measured the baseline serum 25(OH)D and total cholesterol levels of 182 fair-skinned and dark-skinned subjects; and studied the effect of UV radiation on their serum 25(OH)D levels. They found the amount of serum 25(OH)D produced was determined by the amount of cholesterol in the skin, not on skin pigmentation. Matsuoka et al. [23] investigated the effect of racial pigmentation on vitamin D_3_ formation, simulating the process with a fixed dose of UVB radiation and concluded that while racial pigmentation has a photo-protective effect, it does not prevent the generation of normal levels of active vitamin D metabolites. Persons with dark skin also compensate for low 25(OH)D by rapidly converting it to the active 1,25(OH)2D metabolite, thus allowing them to maintain adequate vitamin D status [24]. Skin pigmentation does not appear to negatively affect vitamin D status [25].

Clothing is a barrier to vitamin D photosynthesis but this is an issue only for people who cover themselves from head to toe [26]. It takes relatively little sunlight exposure to acquire adequate stores of vitamin D and few people wear enough clothes to prevent that from happening. Ten to 15 min of sunlight or daylight exposure to a small area of skin (e.g., the forearm or face, etc.) twice a week, without sunscreen, supplies all the vitamin D necessary for health [27]. The belief that sunscreen lotion blocks vitamin D production is based on a 1987 study done by Matsuoka et al. [28] that was funded by the ultraviolet foundation, which is supported by the tanning bed industry. Contradictory information was provided by Diehl and Chiu [29] which concluded that although sunscreens are effective, many may not actually be blocking UV-B because they are improperly or inadequately applied [30]. Thus, sunscreen use may not actually diminish vitamin D synthesis in real world use. However, prolonged unprotected sun exposure should be avoided to reduce the risk of developing skin cancer.

Although pollution can block some ultraviolet radiation, even in urban areas of high pollution 50 % of UV rays reach the ground [31]. A significant amount of UV radiation exposure can be obtained in dense metropolitan areas; tall buildings provide shade but shade gives up to 50 % of UV rays. Indoor workers receive 10–20 % of outdoor workers’ yearly UV exposure [31]; and for many, this may be adequate, especially if sunlight exposure is higher when they are not working. UV radiation is reflected or scattered to varying extents by different surfaces. The scattering and absorption of light by clouds may not significantly reduce natural light exposure because over 90 % of UV rays may penetrate clouds [31]. Environmental factors are rarely an impediment to photosynthesis of adequate vitamin D.

As the skin ages, there is a decline in the cutaneous levels of 7-dehydrocholesterol, resulting in a marked reduction of the skin’s capacity to produce vitamin D_3_ [32]. However, despite the up to fourfold reduction in vitamin D_3_ production in a 70-year-old compared to a 20-year-old, the skin has such a high capacity to make vitamin D_3_ that elders exposed to sunlight will produce an adequate amount of vitamin D_3_ to satisfy their vitamin D requirement [33, 34].

A 1988 study by Webb et al. [35] is often cited to support the conviction that latitude dramatically influences the amount of solar radiation available to synthesize vitamin D_3_. However, other researchers who conducted more recent studies refute this hypothesis. Kimlin et al. [36] report, “It may no longer be correct to assume that vitamin D levels in populations follow latitude gradients”. And Lubin [37] states, “Geophysical surveys have shown that UV-B penetration over 24 h, during the summer months at Canadian north latitudes when there are many hours of sunlight, equals or exceeds UV-B penetration at the equator.” Ross et al. [16] report that ample opportunities exist to form vitamin D (and store it in the liver and fat for later use) from exposure to sunlight during the spring, summer, and fall months even in the far north latitudes.

Acceptance of these vitamin D myths regarding photosynthesis prevents consideration of the alternate hypothesis—low serum 25(OH)D, despite adequate photosynthesis of vitamin D_3_, is a result of an inflammatory process.

## Low vitamin D is found in healthy subjects

**Fig. 2:**
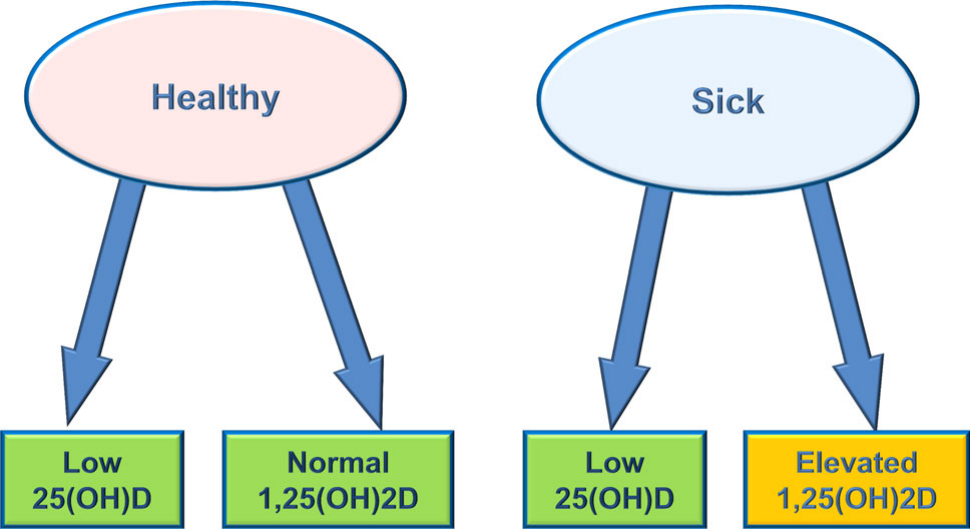
Interpretation of vitamin D deficiency via calcitriol measurement. Since low 25(OH)D is found in both healthy persons and those with autoimmune or chronic inflammatory diseases, assessing vitamin D status with measurement of 1,25(OH)2D may be helpful

Many studies of healthy subjects have found levels of 25(OH)D that, by some vitamin D definitions, are declared deficient (hypovitaminosis-D) [38, 39]. Vitamin D levels that are considered deficient have even been found in persons who are exposed to abundant sunlight [40]. Binkley et al. [41] showed a mean 25(OH)D level of 31.6 ng/ml among healthy young adult Hawaiian surfers. It is clear that low levels of 25(OH)D are found in both healthy persons and those with autoimmune or chronic inflammatory diseases. Opposing reasoning can be used to explain this contradiction. One explanation reasons that healthy persons with low 25(OH)D will become sick and sick people will develop lower 25(OH)D levels (Fig. 2); however, studies do not support this hypothesis. The correct explanation may be that, in the absence of disease, low 25(OH)D is normal.

### Low vitamin D in the presence of diseases

Since low 25(OH)D is found in both healthy persons and those with autoimmune or chronic inflammatory diseases, assessing vitamin D status with the measurement of an additional clinical marker may be helpful. It is asserted that low levels of 25(OH)D accurately reflect vitamin D status; however, measurement of 1,25(OH)2D often demonstrates a positive correlation of elevated 1,25(OH)2D to inflammatory diseases (Fig. 2). This is illustrated by Blaney et al. [42] in a study of 100 patients with autoimmune and chronic disease which found that 85 % of subjects had levels of 1,25(OH)2D higher than 46.2 pg/ml without hypercalcemia. Although this serum level may be considered normal by some, lab ranges for 1,25(OH)2D may have been skewed high by the presence of patients with unrecognized persistent intracellular infection and thus, dysregulated vitamin D metabolism. The Danish 1,25(OH)2D population data (from a large and reliable study) found the mean value for 1,25(OH)2D in a normal population was 29 pg/ml with a standard deviation of 9.5 [43]. More frequent measurement of both D-metabolites in the clinical and research settings, may shed light on the true meaning of low 25(OH)D.

Rickets is often cited as proof of the need for vitamin D supplementation. However, a review of the metabolic processes involved provides some prospective. Adequate vitamin D is essential to prevent rickets, but adequate calcium is equally important; if either calcium or vitamin D is deficient, bone health suffers. Hypophosphatemia is the common denominator of all rickets; low calcium intake leads to hyperparathyroidism, which leads to high phosphorus excretion and, thus, phosphorus deficiency [44]. Rickets is rare in the developed world; however, children in developing countries, who usually photosynthesize enough vitamin D from sunlight, develop rickets if poverty prevents them from eating enough calcium-rich food [45, 46]. Studies have found that rickets occurs in sunny countries due to poor calcium intake and is cured with increased calcium ingestion [47, 48].

Osteoporosis is another disease which is closely linked with vitamin D. Adequate vitamin D is an important factor in maintaining bone health to avoid osteoporosis but a study by Reid et al. [49] published in The Lancet found little evidence supporting the use of vitamin D supplements by seniors hoping to improve bone density and ward off potential fractures. 1,25(OH)2D maintains calcium homeostasis between blood, cells and bones by stimulating calcium absorption from the intestines, reabsorption in the kidneys, and resorption in bones [50]. 1,25(OH)2D up-regulates the VDR in the small intestine, which then transcribes genes that shuttle calcium and phosphorus through the intestinal epithelium. However, mucosal response and calcium/phosphorus absorption are dependent on a competent VDR and elevated 1,25(OH)2D reduces VDR competence [51]. Thus, calcium and phosphorus absorption may be inhibited if VDR function is impaired by elevated 1,25(OH)2D. This is illustrated by Abreu et al. [52] in a study of Crohn’s patients with elevated 1,25(OH)2D and low bone mineral density which concluded that treatment of the underlying inflammation would improve metabolic bone disease. In fact, there is ample evidence that elevated 1,25(OH)2D leads to bone loss. Brot et al. [53] found that elevated levels of 1,25(OH)2D were strongly associated with decreased bone mineral density and content, and increased bone turnover. When levels are above 42 pg/ml 1,25(OH)2D stimulates bone osteoclasts. This leads to osteoporosis, dental fractures and calcium deposition into the soft tissues [54]. Vanderschueren et al. [55] found that a combination of high 1,25(OH)2D and low 25(OH)D is associated with the poorest bone health.

### Vitamin D supplementation

It is reasoned that if low 25(OH)D indicates a current or potential disease state, then increasing 25(OH)D by supplementing with vitamin D should provide some symptom relief and/or protection. So far, there is scant evidence for this hypothesis [56, 57]. According to Ross et al. [16] in the 2010 IOM report, “Outcomes related to autoimmune disorders, cancer, cardiovascular disease and hypertension, diabetes and metabolic syndrome, falls and physical performance, immune functioning, infections, neuropsychological functioning, and preeclampsia could not be linked reliably with calcium or vitamin D intake and were often conflicting.” Despite the recent increase in vitamin D supplementation, chronic diseases have increased and are expected to continue increasing [58, 59].

Consequently, more vitamin D experts are beginning to reconsider vitamin D supplementation among the general population [60]. Recommending higher vitamin D intake to large populations carries the potential risk of overdosing certain individuals [61]. It is difficult to ingest too much vitamin D from food, and natural mechanisms regulate the amount of vitamin D_3_ photosynthesized from sunlight [62]. However, elevated 25(OH)D and hypervitaminosis-D can occur due to vitamin D supplementation [63]. A study by Noordam et al. [65] cast doubt on the causal nature of previously reported associations between low levels of vitamin D and age-related diseases and mortality. A comprehensive review by Autier et al. [65] concluded that low concentrations of 25(OH)D are most likely an effect of health disorders and not a cause of illness. Commenting on the findings in a press statement, Autier et al. [64] advised against vitamin D supplementation and explained the observed discrepancy between observational and randomized trials:

Decreases in vitamin D levels are a marker of deteriorating health. Ageing and inflammatory processes involved in disease occurrence and clinical course reduce vitamin D concentrations, which would explain why vitamin D deficiency is reported in a wide range of disorders. We postulate that inflammation is the common factor between most non-skeletal health disorders and low 25(OH)D concentrations. Inflammatory processes involved in disease occurrence and clinical course would reduce 25(OH)D, which would explain why low vitamin D status is reported in a wide range of disorders. However, increases in 25(OH)D have no effect on inflammatory processes or on disorders at the origin of these processes.

A 2014 meta-analysis by Bolland et al. [66] on the effects of vitamin D supplementation on skeletal, vascular, or cancer outcomes concludes that vitamin D supplementation with or without calcium does not reduce skeletal or non-skeletal outcomes in unselected community-dwelling individuals by more than 15 %. The authors further state that future trials with similar designs are unlikely to alter these conclusions. Because of emerging concerns about elevated 25(OH)D, the IOM has shifted the paradigm from thinking about ‘more is better’ to a more risk-averse approach [67]. It has also challenged the notion that harm should be viewed in terms of vitamin D toxicity such as hypercalcemia, hypercalciuria, or metastatic calcification and has advanced the concept of ‘harm’ in terms of chronic disease outcomes and mortality [16]. Because adverse effects of vitamin D supplementation may take decades to be realized, clinicians (mindful of the medical ethics precept “First, do no harm”) should err on the side of caution; follow the IOM guideline and wait for the results of long-term vitamin D studies.

### Bacterial pathogenesis of low vitamin D hypothesis

**Fig. 3:**
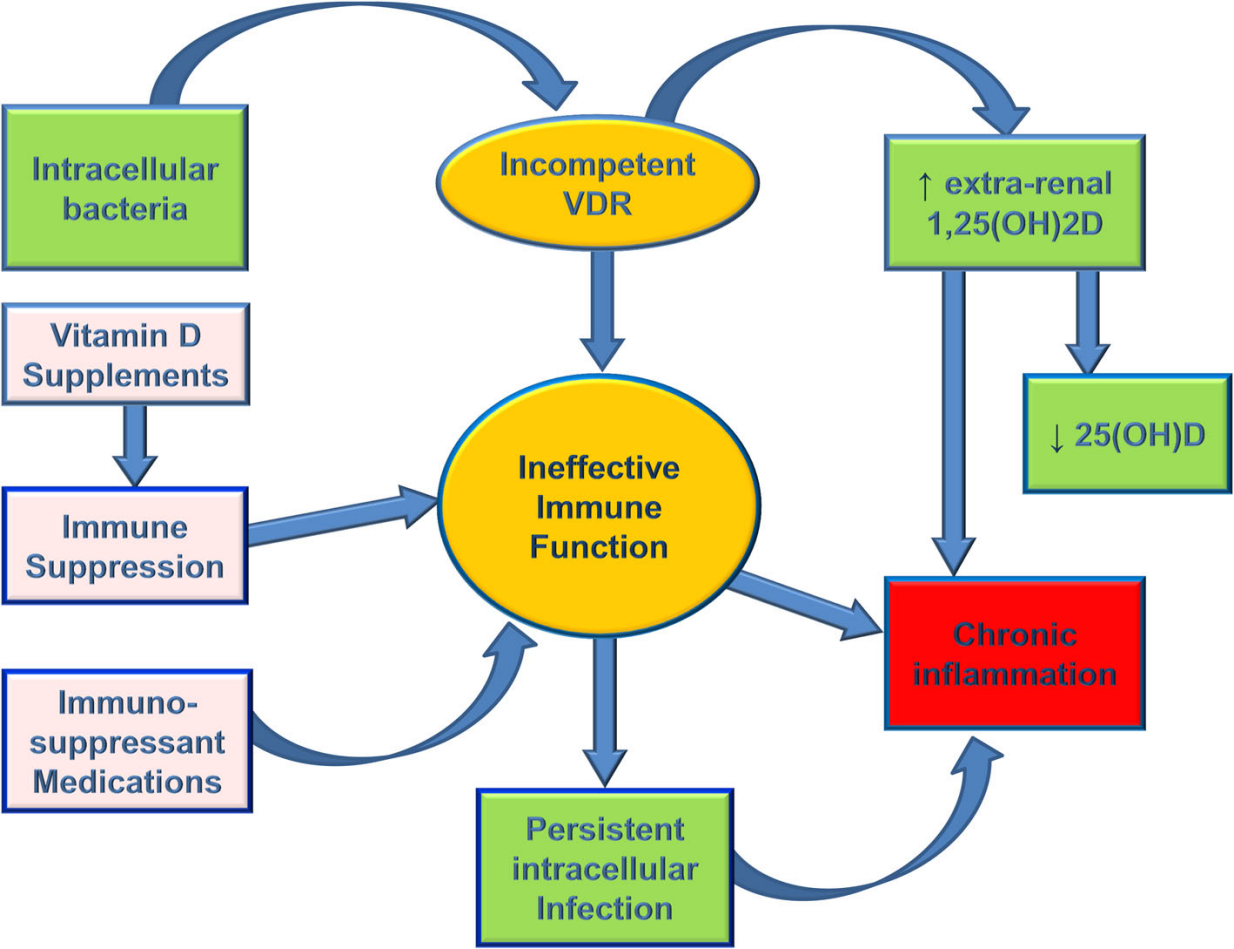
Proposed hypothesis for chronic inflammation caused by persistent intracellular infection. Intracellular bacteria invade nucleated cells and use strategies to avoid destruction. Excess 1,25(OH)2D is produced in an effort to up-regulate the vitamin D receptor to transcribe AMPs; and 25(OH)D is rapidly metabolized in the process, resulting in a low serum level. The resulting elevated 1,25(OH)2D causes chronic, systemic inflammation and its accompanying symptoms

If evidence indicates that most people get adequate vitamin D from sunlight exposure but healthy persons are found to be ‘deficient’ by recent standards, what is the explanation for this phenomenon? Vitamin D proponents use a disease deficiency model to explain low levels of 25(OH)D. Their hypothesis states low 25(OH)D causes chronic diseases; however, a pathogenesis has not been elucidated [68]. Low serum 25(OH)D in the presence of disease can also be explained with a dysregulated vitamin D metabolism model [69]. This hypothesis proposes that low vitamin D is the consequence of a chronic inflammatory process caused by persistent infection. The bacterial pathogenesis theorizes that intracellular (cell wall deficient) bacteria invade nucleated cells, use strategies to avoid destruction and cause abnormal vitamin D endocrine function, resulting in low vitamin D. Excess 1,25(OH)2D is produced in an effort to up-regulate the VDR to transcribe AMPs; and 25(OH)D is rapidly metabolized in the process, resulting in a low serum level. The resulting elevated 1,25(OH)2D causes chronic, systemic inflammation and its accompanying symptoms (Fig. 3).

The existence of bacteria which are capable of invading human cells has been known for over a century and are described by many authors [70, 71]. The lack of a cell wall enables them to enter human cells and proliferate because they fail to elicit an appropriate response when the immune system is compromised. In particular, they enter the macrophages—the very immune cells deployed to kill invading pathogens. The inability of most research labs to culture cell wall deficient (CWD) bacteria has been an obstacle to their acceptance, and reliance on Koch’s postulates has made it difficult to correlate CWD bacteria to specific diseases [72]. But some researchers believe Koch’s postulates may have to be redefined in terms of molecular data when dormant and non-culturable bacteria are implicated as causative agents of mysterious diseases [73]. O´Connor et al. [74] (in their article entitled Emerging Infectious Determinants of Chronic Diseases) report, “microbes can now be irrefutably linked to pathology without meeting Koch’s postulates” and “…powerful tools of molecular biology have exposed new causal links by detecting difficult-to-culture and novel agents in chronic illness settings.”

Domingue [75] commented, “This might translate into an etiology for chronic inflammatory diseases, when the stressed bacteria increase in numbers and overwhelm the normal biological functions of the host.” Nunez [76] was quoted in the University of Michigan Health System newsletter, “In our study, the presence of bacterial microbes inside the cell is what triggers the immune response.” Rolhion and Darfeuille-Michaud [77] observed that the presence of pathogenic invasive bacteria could be the link between an innate immune response to invasive bacteria and the development of inflammation. A number of studies suggest disease associations with CWD bacteria [78–83]. Verway et al. [79] report “…data suggest that at least a subset of the genetic predisposition to Crohn’s disease results from defects in the detection and/or processing of intracellular pathogens by the innate immune system.” O’Connor et al. [74] state, “The epidemiologic, clinical, and pathologic features of many chronic inflammatory diseases are consistent with a microbial cause. Infectious agents likely determine more cancers, immune-mediated syndromes, neurodevelopmental disorders, and other chronic conditions than currently appreciated.”

CWD bacteria are considered communicable but not contagious; protective immunity depends on an effective cell-mediated immune response [80]. It is now well appreciated that pathologic processes caused by infectious agents may only emerge clinically after an incubation of decades [81]. Among the speculated causes of the increase in chronic infections are overuse of beta-lactam antibiotics [82, 83] and immunosuppression via excess 25(OH)D production [84] or immunosuppressive medications [85]. Many microbiologists now believe at least some, if not all, of the inflammation which drives the chronic disease process is caused by the presence of these stealthy intracellular pathogens [86]. A considerable body of experimental and clinical evidence supports the concept that difficult-to-culture and dormant bacteria are involved in latency of infection and that these persistent bacteria may be pathogenic [73, 87, 88]. McDougal [89] states, “Evidence now confirms that non-communicable chronic diseases can stem from infectious agents.”

## Effects of intracellular pathogens on the immune system

Pathogens gain many advantages by parasitizing immune cells and altering nuclear receptor activity. Tissue invasion provides a privileged niche with access to host protein and iron, sequestration from immune response, and a means for persistence [90]. In the arms race of host–microbe co-evolution, successful microbial pathogens have evolved innovative strategies to evade host immune responses. For example, ‘crosstalk manipulation’ undermines host defenses and contributes to microbial adaptive fitness [91, 92]. Pathogenic microbes also induce stress responses which protect the cell from lethal factors, express proteases that degrade AMPs, use biofilms as a shield and modulate host cell motility to facilitate establishment of an infection [93, 94]. Genetic foreign and host protein interactions alter gene transcription and translation mechanisms, and many species survive by horizontal gene transfer [95, 96].

It is theorized that bacteria have developed some of these strategies in order to invade host cells and remain undetected within cellular cytoplasm. Many bacterial pathogens form antibiotic-tolerant persister cells which can replicate within macrophages. In this form they can cause subclinical infection and have been associated with chronic diseases [95, 97, 98]. Intracellular bacteria can modulate cytokine production [99]; and in monocytes and macrophages, cytokine activation markedly inhibits 1,25(OH)2D/VDR gene transcription [100].

Macrophage microbicidal mechanisms are responsible for the control and elimination of pathogens. 1,25(OH)2D production and action in macrophages activates toll-like receptors to increase expression of the AMP cathelicidin which kills infectious invaders [101, 102]. When the immune system is fighting a persistent microbe, inflammatory molecules are continuously released in an effort to kill the pathogen [103]. Immune defenses stimulate Th_17_ cells and contribute to the development of chronic inflammatory conditions [104, 105]. An ineffective immunological response causes low-grade inflammation and phagocyte-inflicted tissue damage plays an important role in many chronic diseases [106]; autoimmune patients acquire a distinct pathogenic microbiota and multi-morbidity often results [107, 108]. Therefore, it is reasonable to infer that bacteria have evolved strategies which allow them to persist within host cells. The exact mechanisms are unknown and warrant further study.

### The compromised immune system, infection and vitamin D

In an essay on the renin–angiotensin system (RAS) and immune response, Smith [109] postulated that unresolved cellular stress may be caused by infectious agents to avoid adaptive immune responses. The host immune response has developed many mechanisms to neutralize and remove pathogenic bacteria. In turn, pathogenic bacteria have developed mechanisms to alter and evade the host immune response [110, 111]. Regulation of the VDR is a common mechanism used in the host defense against pathogens but certain microbes have been shown to slow innate immune defenses by down-regulating the VDR:

- Mycobacterium tuberculosis down-regulates VDR activity [112].
- Mycobacterium leprae inhibits VDR activity through down-regulation of CYP27B1 in monocytes [113].
- Aspergillus fumigatus secretes a toxin capable of down-regulating the VDR in macrophages [114].
- Epstein–Barr virus lowers VDR activity [115].
- HIV completely shuts down VDR activity [116].
- In VDR knockout mice, a circumstance that closely mimics extreme VDR dysregulation, 1,25(OH)2D levels increase by a factor of ten [117].

Studies also point to immune system depression and elevated 1,25(OH)2D in chronic diseases [118]:

- Sarcoidosis patients are deficient in cathelicidin despite healthy vitamin D_3_ levels [119].
- 1,25(OH)2D is high (>60 pg/ml) in 42 % of Crohn’s patients and the source of the active vitamin D may be the inflamed intestine [52].
- 1,25(OH)2D is elevated in the synovial fluid of patients with RA (rheumatoid arthritis) [120].
- Crohn’s disease decreases expression of cathelicidin [121].

High levels of 1,25(OH)2D may result when down-regulation of the VDR by bacterial ligands prevents the receptor from expressing enzymes necessary to keep 1,25(OH)2D in a normal range [42]. Elevated 1,25(OH)2D further reduces VDR competence, suppresses macrophage function, and blocks the nuclear factor kappa-B pathway; thus inhibiting immune system function [116, 122, 123]. Reducing the ability of the VDR to express elements of innate immune function allows intracellular bacteria to persist in the cytoplasm of nucleated cells and may account for the increased susceptibility to non-bacterial co-infections that are commonly found in patients with chronic illnesses [124, 125]. Theoretically, immune system suppression allows parasitic microbes to persist and proliferate in host phagocytes, successfully compete for nutritional resources, and displace commensal organisms from their niche [126]. Elevated 1,25(OH)2D appears to be evidence of a disabled immune system’s attempt to activate the VDR to combat infection.

## Autoimmune disease

Numerous examples can be found in which pathogens express antigens that cross-react with host antigens or induce local inflammatory responses that can lead to autoimmune responses through a very complex set of circumstances [127]. The prevailing theory regarding the etiology of autoimmune disease states that an overactive immune system produces auto-antibodies against self, but infection as an environmental factor in autoimmunity has long been recognized. An alternate hypothesis posits a bacterial etiology in which a persistent intracellular infection causes a cytokine release that induces signals to T cells and B cells, and the antibodies they produce (to the intracellular invader) include some that attack human proteins, as well as target the pathogens [128, 129]. Christen et al. [130] explored this hypothesis, “In theory, a structural similarity or identity between the host and an invading pathogen might cause the immune system of the host to react not only to the pathogen but also to self-components.” Infections can act as environmental triggers inducing or promoting autoimmune disease in genetically predisposed individuals [131]; researchers have shown how antinuclear antibodies (ANA) are created in response to infectious agents [132, 133].

Vitamin D appears to have a positive effect on autoimmune disease due to immune system suppression [122, 134, 135] and immune suppression is considered therapeutically beneficial for autoimmune diseases [136, 137]. However, vitamin D proponents have failed to recognize that positive effects are due to the immunosuppressive effect of elevated 25(OH)D or to understand that immunosuppression is contraindicated because of the probable presence of intracellular infection. When the immune system is suppressed clinical disease markers and symptoms are reduced but immunosuppression does not address an underlying cause of persistent bacteria, thus relapse is common [138]. Verway et al. [79] wonder, “Is a specific pathogen responsible for disease or rather is a dysregulated immune response generated against a complex microbial population? Why would immune-suppressive drugs be efficacious if the primary defect is an immune deficiency?” Much of current research focuses on finding drugs to suppress inflammation but, according to Collins [139], 95 % of these studies have failed It seems clear a better direction is needed. Immunotherapy which restores VDR competence corrects dysregulated vitamin D metabolism and eliminates intracellular bacteria could be the answer (as discussed in the section titled Restoring VDR Competence).

## Dysregulated vitamin D metabolism

**Fig. 4:**
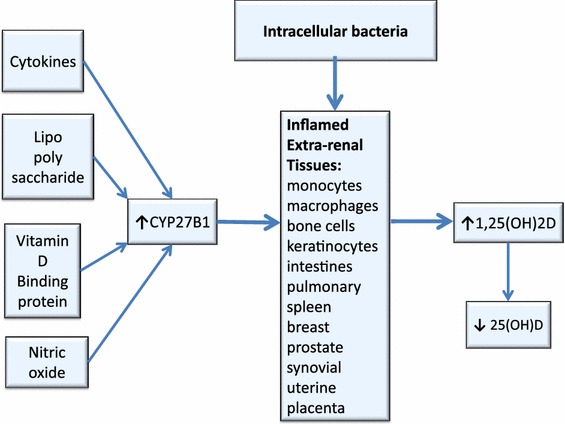
Proposed hypothesis for excess 1,25(OH)2D production in bacterially-stimulated extra-renal tissues. Extra-renal tissues, which produce 1,25(OH)2D, are regulated by cytokines, lipopolysaccharide, nitric oxide and intracellular VDBP, which activate the enzyme CYP27B1 to stimulate conversion of 25(OH)D to 1,25(OH)2D, resulting in low 25(OH)D

In a healthy individual, the complex interplay between innate and adaptive immunity cooperates to mount an appropriate response to infection through regulation of the vitamin D endocrine system [140]. The immune system detects and responds to the presence of intracellular bacteria by producing more 1,25(OH)2D to activate the VDR and express the crucial endogenous AMPs which enable the innate immune system to target intracellular pathogens [141]. Renal production of 1,25(OH)2D is tightly self-regulated, with the end product down-regulating its own further production. In contrast, extra-renal tissues (e.g., uterine decidua and placenta, colon, breast, prostate, spleen, bone, keratinocytes, melanoma and synovial cells, pulmonary monocytes and macrophages, etc.) which produce 1,25(OH)2D are regulated by cytokines (e.g., interferon-gamma), lipopolysaccharide, nitric oxide and intracellular VDBP, which activate the enzyme CYP27B1 to stimulate conversion of 25(OH)D to 1,25(OH)2D [142]. This extra-renal production of 1,25(OH)2D in tissues infected with intracellular bacteria can result in an excess in production of 1,25(OH)2D which may contribute to depletion and low levels of 25(OH)D [143] (Fig. 4).

Because extra-renal production of 1,25(OH)2D is primarily dependent on the availability of 25(OH)D [144], supplementation with vitamin D to increase 25(OH)D may promote the production of 1,25(OH)2D in non-renal tissues that are sites of intracellular infection and result in hypervitaminosis-D. Sunlight appears to play a part in this process and many patients with autoimmune disease report sun sensitivity. The skin (dermal fibroblasts and keratinocytes possess VDR) has the capacity to synthesize 1,25(OH)2D, and represents an important target tissue for 1,25(OH)2D [145]. If keratinocytes in the skin are infected, natural regulation of photosynthesis may be thwarted and solar energy may overstimulate cellular activity, resulting in an increase in cutaneous production of vitamin D_3_, 25(OH)D and 1,25(OH)2D following sun exposure.

**Fig. 5:**
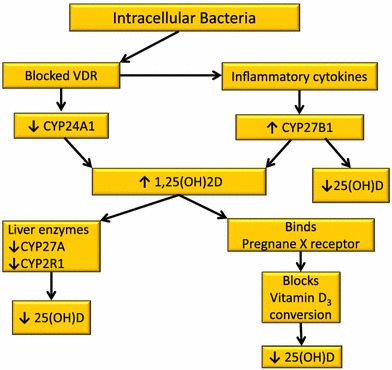
Proposed hypothesis for dysregulated vitamin D metabolism caused by intracellular pathogens. Theoretically, when nucleated cells are parasitized by intracellular bacteria, extra-renal production of 1,25(OH)2D increases, the kidneys lose control of 1,25(OH)2D production, and pro-hormone 25(OH)D decreases due to rapid conversion to 1,25(OH)2D

We hypothesize that when nucleated cells are parasitized by intracellular bacteria, extra-renal production of 1,25(OH)2D increases, the kidneys lose control of 1,25(OH)2D production, and pro-hormone 25(OH)D decreases due to rapid conversion to 1,25(OH)2D. The following mechanisms are thought to be responsible (Fig. 5):

- Inflammatory cytokines activate CYP27B1, an enzyme that causes more 25(OH)D to be converted to 1,25(OH)2D [146].
- The microbial-repressed VDR cannot transcribe CYP24A1 (formerly 24-hydroxylase), an enzyme that breaks down excess 1,25(OH)2D [147].
- Excess 1,25(OH)2D binds the PXR (pregnane X receptor), to inhibit conversion of vitamin D_3_ to 25(OH)D so 25(OH)D is down-regulated [148].
- 1,25(OH)2D inhibits the hepatic synthesis of 25(OH)D [149].

Thus, low 25(OH)D may be a consequence of the inflammatory process. More studies are concluding that suboptimal circulating levels of vitamin D appear to be caused by the disease process. Waldronn et al. [150] found serum 25(OH)D was decreased following an acute inflammatory insult (i.e., orthopedic surgery) and concluded that hypovitaminosis-D may be the consequence rather than cause of chronic inflammatory diseases. Ferder et al. [151] state, “…there may be a relationship between inflammatory processes induced by chronic overstimulation of the renin angiotensin system (RAS) and the worldwide vitamin D deficiency. In fact, the pandemic of vitamin D deficiency could be the other face of increased RAS activity, which could potentially cause a lower activity or lower levels of Vitamin D.”

## Diagnosis of dysregulated vitamin D metabolism

Assessing dysregulated vitamin D metabolism has the potential to guide clinical practice [152, 153]. Vitamin D status is currently determined by measuring the level of serum 25(OH)D which, presumably, reflects the serum levels of other vitamin D metabolites (e.g., vitamin D_3_, vitamin D_2_ and 1,25(OH)2D, etc.). This measurement may not, however, provide enough information to assess vitamin D endocrine function. The clinical utility of measuring 1,25(OH)D is not fully understood, but it is clear that associations are being made between this active metabolite of vitamin D and disease states [154]. 1,25(OH)2D is not being used as a measure associated with vitamin D nutritional status or as an intermediate marker related to health outcomes, or even routinely assessed in vitamin D research. In the context of solving the puzzle of low 25(OH)D, the reasons cited for this lapse fail to consider the possibility of abnormal levels in the presence of chronic inflammation:

- 1,25(OH)2D has a short half-life (hours) and fluctuates rapidly.
  However, a high result may be discovered even at trough level.
- 1,25(OH)2D levels are regulated by PTH, calcium, phosphate.
  This is not true if extra-renal production is prevalent [143].
- 1,25(OH)2D does not decrease until 25(OH)D is very low.
  A low 25(OH)D may be a sign that 1,25(OH)2D is abnormally high [55].
- 1,25(OH)2D is only over-produced in hypercalcemic disease states such as sarcoidosis.
  Studies show this is not true [42].
- 1,25(OH)2D may be elevated as a result of up-regulation of the CYP27B1 enzyme.
  This begs the question, Why is this enzyme elevated [146]?

Measuring both 25(OH)D and 1,25(OH)2D (and PTH, calcium, phosphate when indicated) as clinical markers in chronic disease is more likely to provide a true picture of vitamin D status, than measuring 25(OH)D alone [155, 156] (Table 1). Measuring 1,25(OH)2D should be considered in patients with low 25(OH)D, abnormal laboratory results (especially inflammatory markers), a diagnosis of autoimmune disease or other chronic inflammatory illness, or signs of chronic systemic inflammation. For example, elevated 1,25(OH)2D may serve as a marker of Crohn’s disease [52]. The 1,25(OH)2D test is a delicate assay which is only done in specialized laboratories. False low results have been observed due to apparent sample mishandling; freezing for transport is advised to prevent sample degradation due to agitation. A high result is always accurate.

**Table 1:** D-metabolites tests

Serum 25(OH)D [157]
CPT code: 82306 [157]
Lowest mortality reported at 20 ng/ml [158]
Immunosuppression reported when higher than 30 ng/ml [122]
Serum 1,25(OH)2D [157]
CPT code: 82652 [157]
ICD-9 code:
275.40 Disorder of calcium metabolism, unspecified [157]
Maximum normal = 45 pg/ml [17]

## Restoration of VDR competence

The ability to mount an appropriate immune system response to intracellular infection is highly dependent on a competent VDR [159]. When it appears that 1,25(OH)2D is unable to up-regulate the VDR due to microbial activity, VDR competence may be restored with another VDR ligand which acts as an agonist; an agonist increases the signal transduction activity of a cell when bound to a receptor on that cell. Over 3000 synthetic VDR ligands have been identified, but most of these 1,25(OH)2D analogues have no clinical use because of their undue disruption to calcium regulation [160]. A number of non-vitamin D VDR ligands have also been identified: curcumin, omega-6 fatty acids (e.g., arachidonic acid, linoleic acid), and lithocolic acid (LCA) but are not being used for this purpose [161, 162].

**Fig. 6:**
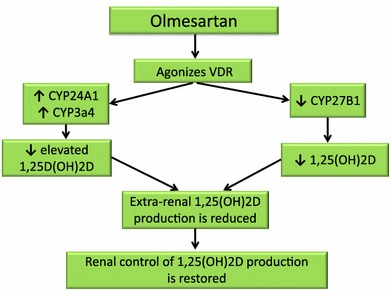
Proposed hypothesis for restoring renal control of 1,25(OH)2D with olmesartan. Olmesartan is believed to decrease elevated 1,25(OH)2D by several mechanisms

Angiotensin receptor blockers (ARBs) have been shown, via in silico molecular modeling, to modulate VDR activation [163]. The most promising ARB, olmesartan medoxomil (brand name Benicar^®^) was estimated to have a Ki value in the low nanomolar range, similar to the Ki values of the natural VDR ligands [163]. Olmesartan has been noted to cause a significant reduction in elevated 1,25(OH)2D within weeks of initiation, which provides further evidence of its ability to up-regulate the VDR [164]. Olmesartan is believed to decrease elevated 1,25(OH)2D by several VDR-mediated effects (Fig. 6). The up-regulated VDR:

- transcribes CYP24A1 and CYP3A4 (enzymes which reduce 1,25(OH)2D production) [147].
- represses CYP27B1 (the enzyme that hydroxylates 25(OH)D to 1,25(OH)2D) so less 1,25(OH)2D is produced [146].

Consequently, renal control of 1,25(OH)2D production is restored and extra-renal production of 1,25(OH)2D is reduced. A decrease in elevated 1,25(OH)2D means less systemic inflammation, as these studies of olmesartan indicate:

- Improvement of glycemic control and insulin resistance was only observed in the olmesartan group and these effects of olmesartan might be mediated by an anti-inflammatory action [165].
- Olmesartan treatment significantly reduced serum levels of inflammatory markers; h-CRP, h-TNFa, IL-6, MCP-1 after 6 weeks of therapy [166].
- Blocking angiotensin-converting enzyme induces potent regulatory T cells and modulates TH1- and TH17-mediated autoimmunity [167].
- Blocking angiotensin II receptor increases bone mass [168, 169].

**Fig. 7:**
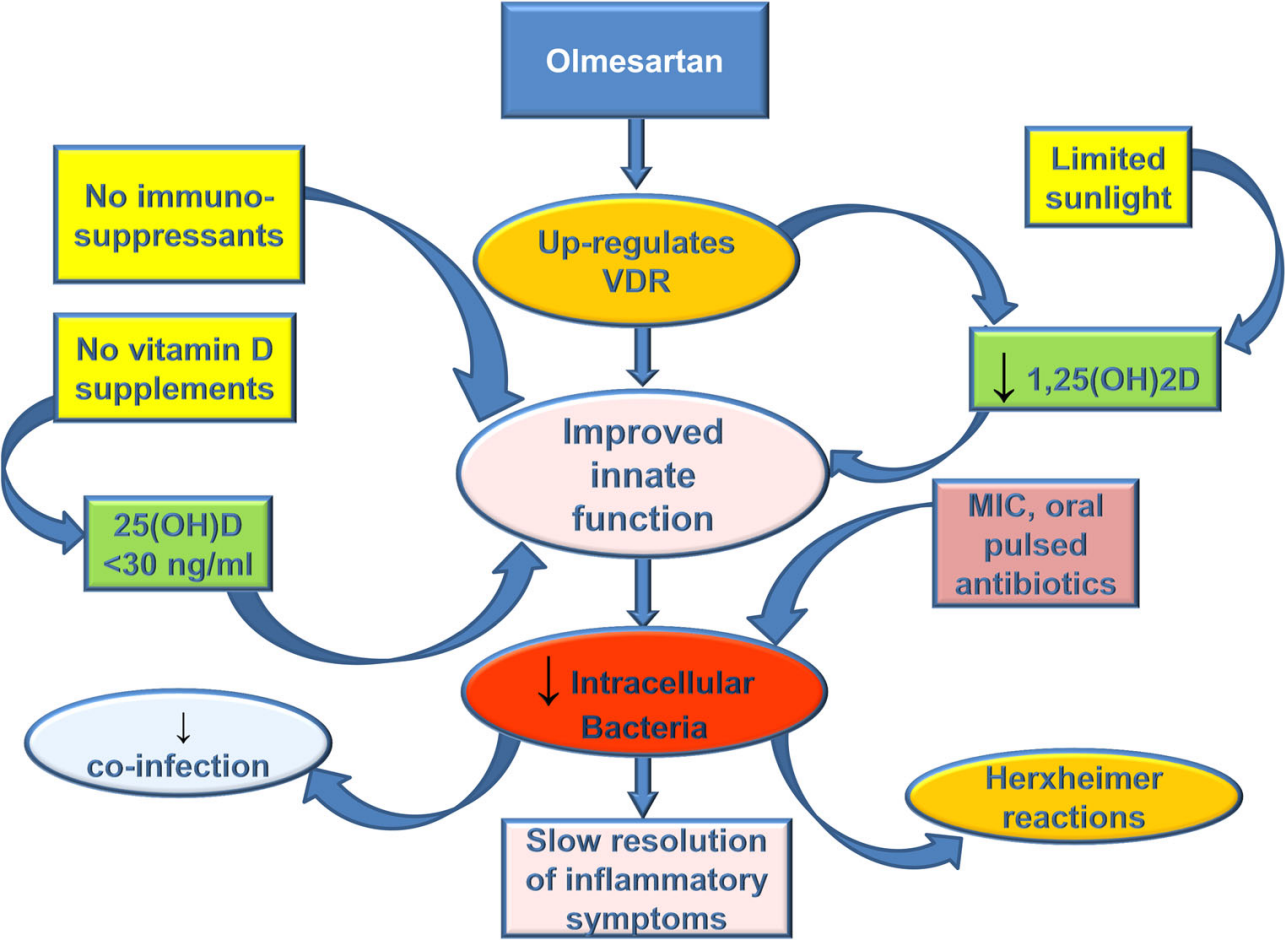
Effect of treatment with olmesartan and antibiotics on inflammatory symptoms. Olmesartan up-regulates the vitamin D receptor to improve innate immune system function and reduce elevated 1,25(OH)2D. Avoidance of immunosuppressants and elevated 25(OH)D also improves immune system function. Inflammatory symptoms gradually resolve as intracellular bacteria are slowly eliminated with the help of select low-dose, pulsed antibiotics

Olmesartan acts in a manner similar to 1,25(OH)2D to reduce inflammation and, by inference, improve immune system function. VDR and RAS receptors are distributed in almost the same tissues. The endogenous VDR ligand 1,25(OH)2D down-regulates the RAS by repressing renin gene expression to reduce inflammation via the nuclear factor-kappa B pathway [170]. Olmesartan has a similar effect in that it reduces angiotensin II (a peptide that is implicated in the inflammatory process) [171]. Inappropriate stimulation of the RAS has been associated with the pathogenesis of hypertension, heart attack and stroke [151]. Ferder et al. [151] state, “Changes in RAS activity and activation of the VDR seem to be inversely related, making it possible to speculate that both systems could have a feedback relationship. The combination of RAS blockade and VDR stimulation appears to be more effective than each one used individually.” Blocking angiotensin II and stimulating the VDR is what olmesartan appears to accomplish and this function is consistent with a theory of VDR incompetence (Fig. 7).

In patients with autoimmune disorders and inflammatory symptoms, olmesartan is noted to provoke an increase in inflammatory symptoms indicative of a Jarisch–Herxheimer reaction (JHR). JHR is a cascade of reactions including inflammation, cytokine release, and endotoxin release as part of the immune response to the disintegration of infected cells [172]. This immunopathology suggests transcription of AMPs by an activated VDR, points to the presence of occult infection and provides additional evidence that olmesartan is a VDR agonist [167, 173, 174]. Theoretically, olmesartan restores VDR competence and, thus, phagocytosis leads to bacterial death; consequently, inflammation is temporarily increased by cytokine reaction to microbial endotoxins and cellular debris from dead host cells and bacteria [175].

Hajishengallis and Lambris [92] conclude that a blockade of hijacked receptors of the innate immune system may offer promising options to control infection and associated immunopathology. Although this use of olmesartan is off-label, its safety profile is well established [176]. The multiple, documented beneficial effects of olmesartan, including the ability to reduce cardiovascular and kidney disease, prevent migraines, and reduce oxidative stress, also suggest it could play a key role in the resolution of chronic systemic inflammation [177, 178].

## Clinical use of olmesartan

Olmesartan is being used as a novel VDR ligand in the clinical setting [179]. Immunotherapy with olmesartan may also include pulsed administration of select MIC (minimum inhibitory concentration) oral antibiotics to weaken and help eradicate the intracellular pathogens. With each antibiotic dose, inflammatory symptoms (JHR) wax and wane, providing further evidence of persistent infection [180]. Changes in laboratory findings (e.g., BUN, creatinine, CRP, blood counts, liver enzymes) often point to areas of occult inflammation. A correlating treatment strategy is the avoidance of excessive sunlight exposure, foods high in vitamin D and vitamin D supplements to maintain serum 25(OH)D at a level (20–30 ng/ml) that is not likely to suppress the immune system and inhibit bacterial elimination [122, 135, 137].

Accumulating case reports now support the observation that a number of complex, chronic conditions can be improved by restoring VDR function using this type of immunotherapy [179, 181, 182]. It is becoming increasingly clear that microbes slow down immune reactivity by dysregulating the VDR, ultimately to increase their chance of survival. Immune modulatory therapies that enhance VDR expression and activity should, therefore, be considered in the clinical setting [183].

## Discussion

Vitamin D is essential for many important biological processes and most people get an adequate supply from exposure to sunlight (Table 2). Long-term studies are needed to determine if low 25(OH)D in healthy individuals leads to disease. Evidence that vitamin D supplementation cures or prevents chronic disease is inconsistent. Despite increased supplementation chronic inflammatory diseases are on the rise. Attention to the alternate hypothesis—low 25(OH)D is a consequence of the chronic disease process, provoked by persistent intracellular infection—may be crucial to reversing this trend and needs further research. The prevailing dogma that the level of serum 25(OH)D provides an accurate assessment of vitamin D status needs closer examination. Circulating levels of 25(OH)D may not be an accurate reflection of vitamin D status. In those with an autoimmune disease or chronic inflammatory symptoms, 1,25(OH)2D may be elevated. This can lead to osteoporosis and cause inhibition of innate immunity, which is contraindicated in the presence of infection. The resulting immunosuppression may promote persistent infection which has been implicated in chronic inflammatory diseases.

**Table 2:** Key points

Vitamin D is a steroid hormone which regulates immune system function
Photosynthesis of vitamin D_3_ provides adequate vitamin D stores for most individuals
Low levels of 25(OH)D are seen in healthy individuals, as well as those with chronic inflammatory conditions
Studies are inconsistent regarding the health benefits of increasing vitamin D stores; vitamin D supplementation may have negative effects
25(OH)D may not always reflect the level of 1,25(OH)D; accurate assessment of vitamin D status depends on measuring both metabolites
Intracellular, cell wall deficient bacteria may cause dysregulated vitamin D metabolism and impaired immune system function
A novel, non-vitamin D VDR ligand (an angiotensin receptor blocker) appears to reactivate the immune system, restore VDR competence, correct dysregulated vitamin D metabolism and reduce inflammatory symptoms

Human cells live in harmony with many types of microbes but some microbes may become pathogenic under commonly experienced conditions. The innate immune system is designed to kill pathogens via 1,25(OH)2D-mediated VDR transcription of anti-microbial peptides but microbes may use strategies which down-regulate the VDR in order to live and reproduce within nucleated host cells. Studies using more advanced cell culture and molecular techniques are confirming the presence of previously undetected intracellular bacteria. Defense mechanisms that intracellular bacteria use to persist and proliferate need to be investigated. Pathogen-induced VDR dysfunction which causes the release of pro-inflammatory cytokines appears to be at the root of chronic disease and low 25(OH)D. Improving VDR activation may be the key to reducing inflammatory diseases. Treatments that up-regulate the VDR to restore normal immune function, reduce inflammation and eradicate persistent bacterial infections require further research. An immunotherapy which has demonstrated efficacy in reversing vitamin D metabolism dysfunction and reducing inflammatory symptoms is currently being used by clinicians and warrants formal study.

In summary, elevated 1,25(OH)2D, often accompanied by reduced 25(OH)D, is a clinical sign of dysregulated vitamin D metabolism and evidence that the immune system is competing with parasitic microbes for VDR dominance. Failure of the immune system to mount an effective anti-microbial response results in persistent intracellular infection. This induces relentless inflammation (immunopathology) which causes tissue damage and disease symptoms.
